# Resilience of beach grasses along a biogeomorphic successive gradient: resource availability vs. clonal integration

**DOI:** 10.1007/s00442-019-04568-w

**Published:** 2019-12-04

**Authors:** Valérie C. Reijers, Carlijn Lammers, Anne J. A. de Rond, Sean C. S. Hoetjes, Leon P. M. Lamers, Tjisse van der Heide

**Affiliations:** 1 Department of Aquatic Ecology and Environmental Biology, Institute for Water and Wetland Research, Radboud University, Heyendaalseweg 135, 6525 AJ Nijmegen, The Netherlands; 2 Department Coastal Systems, Royal Netherlands Institute for Sea Research and Utrecht University, 1790 AB Den Burg, The Netherlands; 3 Conservation Ecology Group, Groningen Institute for Evolutionary Life Sciences, University of Groningen, Groningen, The Netherlands

**Keywords:** Clonal plants, Coastal dunes, Biogeochemistry, *Ammophila arenaria*, *Elytrigia juncea*

## Abstract

Coastal ecosystems are often formed through two-way interactions between plants and their physical landscape. By expanding clonally, landscape-forming plants can colonize bare unmodified environments and stimulate vegetation–landform feedback interactions. Yet, to what degree these plants rely on clonal integration for overcoming physical stress during biogeomorphological succession remains unknown. Here, we investigated the importance of clonal integration and resource availability on the resilience of two European beach grasses (i.e. *Elytrigia juncea* and *Ammophila arenaria*) over a natural biogeomorphic dune gradient from beach (unmodified system) to foredune (biologically modified system). We found plant resilience, as measured by its ability to recover and expand following disturbance (i.e. plant clipping), to be independent on the presence of rhizomal connections between plant parts. Instead, resource availability over the gradient largely determined plant resilience. The pioneer species, *Elytrigia,* demonstrated a high resilience to physical stress, independent of its position on the biogeomorphic gradient (beach or embryonic dune). In contrast, the later successional species (*Ammophila*) proved to be highly resilient on the lower end of its distribution (embryonic dune), but it did not fully recover on the foredunes, most likely as a result of nutrient deprivation. We argue that in homogenously resource-poor environments as our beach system, overall resource availability, instead of translocation through a clonal network, determines the resilience of plant species. Hence, the formation of high coastal dunes may increase the resistance of beach grasses to the physical stresses of coastal flooding, but the reduced marine nutrient input may negatively affect the resilience of plants.

**Electronic supplementary material:**

The online version of this article (10.1007/s00442-019-04568-w) contains supplementary material, which is available to authorized users.

## Introduction

Vegetated coastal ecosystems including coastal dunes, salt marshes and seagrass meadows underpin vital services in coastal zones (e.g. flood protection, water and carbon storage, biodiversity enhancement) (Costanza et al. 1997; Barbier et al. 2008, 2011). The dynamics of these ecosystems are generally controlled by biophysical feedback mechanisms. These landscape-forming feedbacks are generated through two-way interactions between the dominant plant species and physical processes, ameliorating the stressful conditions that typically prevail in exposed coastal environments (Corenblit et al. 2011; Balke et al. 2014; Corenblit et al. 2015a, b; Vacchi et al. 2017). For instance, plants are able to attenuate flows of wind and water, thereby stimulating sedimentation and promoting their own growth by reducing physical stress (e.g. drag, salinity) (Jones et al. 1994; Van Hulzen et al. 2007; van der Heide et al. 2007; Zarnetske et al. 2012; Silliman et al. 2015). Because these feedbacks require a minimum plant shoot density and patch size to operate adequately, feedback-dependent ecosystems can suddenly collapse below this threshold, and (re-)establishment is impeded (Christianen et al. 2014; Silliman et al. 2015; Angelini et al. 2016).

To rapidly overcome these density- and patch size-dependent establishment thresholds, many landscape-forming plants rely on clonal expansion as their main mode of dispersal (Bouma et al. 2005; Kendrick et al. 2005; Hacker et al. 2012, Reijers et al. 2019a). Next to the ability to generate high local shoot numbers, increase patch size and steer shoot organization, clonality can also greatly enhance the longevity of an individual (de Witte and Stöcklin 2010; Thomas 2013; Bricker et al. 2018). This significantly increases its persistence and potential of forming complex biogeomorphic landscapes (Bouma et al. 2013; Strain et al. 2017). The stressful conditions prevailing in exposed bare, unmodified coastal systems (e.g. intertidal mudflats, beach plains) hamper successful establishment of plant species. Expansion into these stressful environments can be facilitated by physiological integration of pioneer shoots with a sufficiently large original patch through rhizomal connections (Amsberry et al. 2000; Silinski et al. 2016). By sharing scarce resources such as water, carbohydrates and mineral nutrients over the clonal network, clonal plants can mitigate stressful or resource-poor conditions (Alpert and Mooney 1986; Alpert 1996; Stuefer et al. 1994; Brewer and Bertness 1996; Pennings and Callaway 2000; He et al. 2011). However, to what degree landscape-forming plants rely on clonal integration throughout the various phases of biogeomorphological succession, from bare unmodified environments to biologically engineered habitat, remains poorly understood (Kendrick et al. 2005; Corenblit et al. 2011, 2015a, b).

Here, we hypothesize that landscape-forming plants rely on clonal integration for overcoming establishment thresholds during the early phases of biogeomorphic succession on bare sediment, but that the significance of clonal integration fades as the landscape becomes increasingly modified through biophysical feedbacks. We tested this general hypothesis in a coastal dune environment on the Western European coast, by studying the role of clonal integration for overcoming physical stress over a natural biogeomorphic succession gradient from beach (unmodified, stressful environment) to foredune (modified, more benign environment).

Coastal dunes are found all over the world in wave-dominated sandy beaches where an ample supply of ocean-provided sediment drives the aeolian transport of sand particles (Martínez et al. 2008). Burial-tolerant beach grasses can trap and accumulate wind-blown sand, thereby forming small embryonic or incipient dunes (van Puijenbroek et al. 2017a, b). In time, these embryonic dunes can develop into foredunes that form a resistant coastal defence line (Hesp 2002; Durán and Moore 2013). As the harsh environmental conditions on the beach (e.g. prolonged inundation, salinity stress, wave exposure) limit plant growth, accreting sediment offers an effective escape mechanism for many dune-building grasses (Baye 1990; Maun 1994). However, by accreting sediment, beach grasses do not only escape from the physical and physiological stresses of living on the land–sea interface, but they also lose the advantage of receiving a higher external nutrient input (e,g. deposit of wrack or other organic matter) during overwash events (Dugan et al. 2011; Schrama et al. 2013). As nutrient levels in coastal dune environments are generally low, this nutrient deprivation may seriously hamper the recovery rate and resilience of the dune-building grasses growing at the crest of the foredune.

In Northwestern Europe, the two main dune-building grasses, *Elytrigia juncea* (L.) Nevski (hereafter *Elytrigia*) and *Ammophila arenaria* (L.) Link (hereafter *Ammophila*), are generally found in subsequent successional stages. *Elytrigia* is the pioneer species that generates large but sparse vegetation patches, thereby rapidly colonizing the unmodified beaches to form wide and low embryonic dunes (van Puijenbroek et al. 2017a, b). *Ammophil*a in contrast is the later successional species that generally colonizes the embryonic dunes to form small, but dense vegetation patches that lead to the formation of narrower but higher foredunes (van Puijenbroek et al. 2017a, b; Reijers et al. 2019a).

Based on our overarching hypothesis, we suggest that for coastal dune systems in general: (1) dune-forming species rely on clonal integration in the early successional phase of beach colonization, but that this effect wears off in later successional phases and (2) exposure to physical stress and resource availability synergistically determine the resilience of beach grasses to severe physical stress. Specifically, for our study system we expect the pioneer species, *Elytrigia,* to rely strongly on clonal integration to rapidly colonize the barren landscape and overcome the environmental harshness of growing at the land–water interface. In contrast, we expect that the later successional species, *Ammophila*, is less adapted to physical stress and does not rely so much on physiological integration between its connected dense tussocks to overcome physical stress. Moreover, as both physical stress and resource availability likely decrease over the successive dune gradient, we anticipate plants growing on the higher end of the species’ spatial distribution to be less resilient to physical stress and to rely less on distributing resources between their clonal networks.

To test both hypotheses, we set up a field experiment over a coastal dune successive gradient on a single location in a wide dissipative dune system on the Dutch barrier coast. We mimicked severe physical stress, which can be caused by storm surges or grazing by rabbits (Feagin et al. 2015; Harris and Davy 1986a), by clipping all aboveground biomass, and monitored the recovery rate and nutrient availability of both species as an indicator of their resilience to physical stress. The effect of clonal integration in overcoming physical stress for both species was tested by leaving the clonal network either intact or by severing the clonal plant into two parts. Specifically, we addressed the following questions: (1) How do both beach grasses respond to severe physical stress? (2) How does the position (high vs. low) on the successive gradient affect the resilience of both beach grasses? (3) Does clonal integration help both beach grasses recover after severe physical stress? (4) Does the importance of clonal integration in overcoming physical stress decrease over a successive gradient when there are fewer resources available?

## Materials and methods

### Study system

**Fig. 1:**
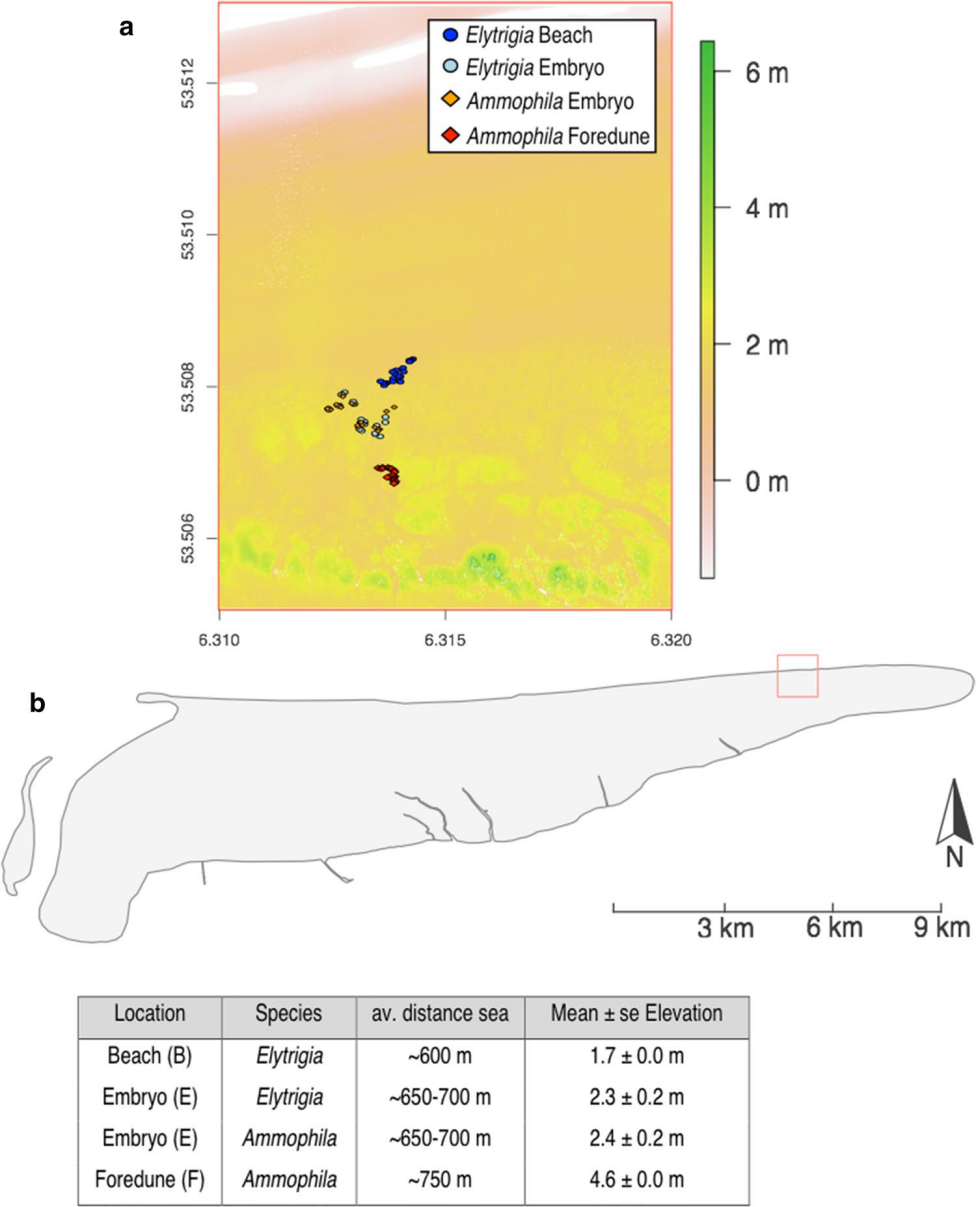
**a** The location of our experimental plots projected on a digital elevation model (DEM) in metre above MWL [obtained from Actueel Hoogtebestand Nederland (AHN) (van der Zon 2013)] of the beach at the eastern end of Schiermonnikoog. The dark blue rounds represent the *Elytrigia* plots located on the beach (B). The light blue round and orange diamonds represent *Elytrigia* and *Ammophila,* respectively, in the embryonic dune system (E). Finally, the red diamonds depict the *Ammophila* plots in the foredune region (F). **b** Topographic characteristics of the four different plot types. The full colour version of this figure is available online

The study area was located on a wide dissipative beach on the eastern end of the Wadden Sea island of Schiermonnikoog, the Netherlands (53° 30′ 27 N, 6° 18′ 40 E). With a mean tidal range of 2.2 m, the island is characterized as a mesotidal barrier island (Osté et al. 2013). The beach width of ~ 750 m, as measured from the 0 mean water level (MWL) line to the primary foredunes, is wide enough to support large embryonic dune complexes (van Puijenbroek et al. 2017a, b). The two dominant beach grasses of our study system, sand couch (*Elytrigia juncea*) and marram grass (*Ammophila arenaria*), differ in their spatial distribution with *Elytrigia* occupying the lower ranges of the coastal dune gradient (i.e. beach and embryonic dunes) and *Ammophila* occupying the higher ranges from the embryonic dunes to the high foredunes. With an average height of 1.7 m above HWL, the beach region, where only *Elytrigia* occurs, is likely to get partially flooded every springtide (Osté et al. 2013) (Fig. 1). The embryonic dune region where both species can be found is situated ~ 60 cm higher and will get flooded approximately five times a year during storm surges (primarily during winter time) (Osté et al. 2013). Finally, the higher region of the coastal foredune (+ 4.6 m HWL) is unlikely to receive seawater intrusion during coastal overwash events.

### Experimental design

To examine the relative importance of clonal integration in overcoming stress over a successive coastal dune gradient, we selected pairs of individual plants growing in close proximity (approximately within 5 m) and either left their clonal network intact or severed their rhizomal connections. Over a successive transect from beach to foredune, we chose three different locations [beach (B), embryonic dune (E) and foredune (F)], and on each location we selected ten pairs of isolated individuals of either sand couch (*Elytrigia juncea*) or marram grass (*Ammophila arenaria*) at the start of the growing season in early April 2017.

On the lowest end of the gradient on the beach (B), we randomly selected ten pairs of clonal *Elytrigia* individuals (number of shoots: 15 ± 1.2; 0.82 ± 0.10 g FW; *N* = 20). In the embryonic dune region (E) the species were co-occurring and we selected both ten pairs of *Elytrigia* individuals (number of shoots: 43 ± 3; 1.50 ± 0.11 g FW; *N* = 20) and ten paired plots of *Ammophila* individuals (number of shoots: 90 ± 10.3; 4.04 ± 0.28 g FW; *N* = 20). On the highest end of the gradient (F) only *Ammophila* was present (number of shoots: 83 ± 7.2; 3.58 ± 0.26 g FW; *N* = 20) (see Fig. 1). We created our experimental plots (60 × 30 cm) using a frame with sharp edges, which severed all rhizomal connections growing outside of the plot dimensions. Furthermore, if necessary, we removed all surrounding vegetation from a 50 cm distance of the plots.

Of each pair (*N* = 10), one of the plots was randomly chosen to receive the severed clonal network treatment. This experimental design yielded eight treatment combinations: *Elytrigia* intact beach (EIB), *Elytrigia* severed beach (ESB), *Elytrigia* intact embryo (EIE), *Elytrigia* severed embryo (ESE), *Ammophila* intact embryo (AIE), *Ammophila* severed embryo (ASE), *Ammophila* intact foredune (AIF) and *Ammophila* severed foredune (ASF), with ten replicates per treatment. For each experimental plot we excavated the network in the middle of the plot to make sure that the shoots on either side of the plot were linked through rhizomal connections. For the severed clonal integration treatment, we disrupted the rhizomal network in the middle of the experimental plot, using a sharp blade, thereby dividing the clonal individual into two separate parts (labelled part 1 and part 2). For both the severed and the intact clonal treatment, we used wooden poles to mark the division between both parts of the plant. At the start of the experiment, we clipped all aboveground biomass of all our experimental units to mimic severe physical stress. In this way we could investigate the role of clonal integration in overcoming stress over a successive gradient for both species. The experiment lasted for a total of 62 days.

### Soil analysis

To evaluate the potential differences in nutrient availability over the successive coastal dune gradient, we collected sediment samples at the beginning of the experiment in early April 2017. Sediment samples were collected (~ 10 cm depth), from both inside the plot between the plant roots, and at 50 cm from the edge of the plant. Salt extracts were taken from the soil samples using 17.5 g fresh soil in 50 ml of 0.2 M NaCl, which was shaken for 2 h. Plant-available nitrogen was estimated by colorimetrically measuring concentrations of nitrate and ammonium in the salt extracts on an AutoAnalyzer 3 system (Bran and Luebbe, Norderstedt, Germany or Skalar and Seal autoanalyzer). Nitrate was determined by sulphanilamide, after reduction of nitrate to nitrite in a cadmium column (Wood et al. 1967) and ammonium using salicylate (Grasshoff and Johannsen 1972). Plant-available phosphorus was estimated using a bicarbonate extract (Olsen 1954), which was analysed using an inductively coupled plasma emission (ICP) spectrophotometer (ICP-OES iCAP 6000; Thermo Fisher Scientific, Waltham, MA, USA).

### Plant analyses

To calculate the rates of recovery and expansion of the different plant treatments, we counted the number of shoots of each experimental plot at the beginning, after 21 days and at the end of the experiment after 62 days. To determine the foliar nutrient and sodium concentrations as proxies for nutrient uptake and marine influence, we collected leaf samples of either side of the experimental plots (part 1 and part 2) both at the beginning of the experiment and at the end, yielding a total of 320 leaf samples (80 experimental units × 2 part per plot × 2 time points). After drying at 60 °C to constant weight, we ground the samples using a ball mill (MM400, Retch, Haan, Germany). Subsequently, to determine C and N concentrations we weighed ~ 1 mg homogenized samples in tin cups and analysed them using an elemental analyser (Carlo Erba NA1500, Thermo Fisher Scientific, Waltham, MA, USA). Furthermore, the concentrations of P and Na were determined on 200 mg of the aboveground plant material through digestion with 4 ml of HNO_3_ (65%) and 1 ml of H_2_O_2_ (30%) in a microwave oven (MLS 1200 Mega, Milestone Inc., Sorisole, Italy), after which the samples were diluted and analysed using an inductively coupled plasma emission (ICP) spectrophotometer (ICP-OES iCAP 6000; Thermo Fisher Scientific, Waltham, MA, USA).

### Statistical analyses

All statistical analyses were performed using the software program R (version 3.4.0). The main and interactive effects of clonal integration treatment (severed vs. intact), species (*Elytrigia* vs. *Ammophila*) and their position on the biogeomorphic gradient (low vs. high per species) per time point (*t* = 21 and *t* = 62 days) on the recovery rate of the individual plants for both time points were analysed using linear mixed effect models with a Satterthwaite approximation of the degrees of freedom using the pairs as a random factor. For soil nutrient values, we used linear mixed effect models to assess the main effects of both position on the biogeomorphic gradient (beach, embryonic dune, foredune) and vegetation presence (within patch vs. outside patch) on both plant-available N and P levels, using the pairs as a random factor. Tukey HSD post hoc tests were used to separate treatment effects.

The main and interactive effects of species identity (*Elytrigia* vs. *Ammophila*), position on the biogeomorphic gradient (low vs. high per species) and time of measurement (start vs. end of the experiment) on foliar nutrient levels (two samples per plant) were analysed using linear mixed effect models with a Satterthwaite approximation of the degrees of freedom using plot number as a random factor. To quantify the variation in nitrogen content (Var N) between the two plant parts (*N*_1_ and *N*_2_) as a proxy for clonal integration, we calculated the distance in the ratio between plant parts from a perfect 1:1 relation (1):

1 $${\text{Var N}} = \left( {\frac{{N_{1} }}{{N_{2} }} - 1} \right)^{2}$$

The results are given in (Online Resource 1 Table S1). For every test, normality of the residuals was checked and, if needed, the data were transformed using a square root or Box–Cox transformation. *P* values lower than 0.05 were considered statistically significant.

## Results

### Recovery rates of both beach grasses

**Fig. 2:**
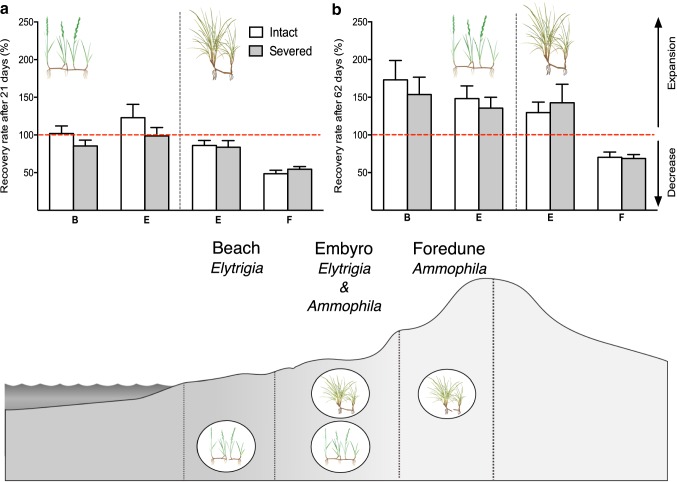
The relative recovery rate of both beach grasses crossed with both clonal integration treatments along the successive gradient from beach (B), embryonic dune (E) to foredune (F) after **a** 21 days and **b** 62 days. *Elytrigia* is always depicted on the left of the black dashed line and *Ammophila* on the right. The red horizontal dashed line indicates the 100% recovery line: above this line the plants have expanded compared to the start of the experiment, below the line the plants have decreased in shoot numbers. Error bars represent + SE

After 21 days, we observed a clear species-specific response to physical disturbance (i.e. clipping of shoots), with almost all *Elytrigia* plants showing full recovery, whereas 40% of *Ammophila* plants had not yet recovered [101.6 ± 6.1% (*Elytrigia*) vs. 68 ± 4.0% (*Ammophila*); *F*_1,71_ = 27.01; *P *< 0.001; Fig. 2a]. Strikingly, we observed no significant differences between clonal integration treatment (intact vs. severed) for either species (*F*_1,71_ = 0.71; *P *= 0.403). We did find a clear interaction between the position on the biogeomorphic gradient and the species, with *Ammophila* showing less recovery on the foredune (51.5 ± 2.9%) than on the embryonic dunes (84.9 ± 5.3%), whereas *Elytrigia* showed a slightly better recovery in the embryonic dunes (110.2 ± 10.4%) than on the beach (93.6 ± 6.5%) (*F*_1,71_ = 17.42; *P *< 0.001).

At the end of the experiment (62 days after clipping of all aboveground biomass), all *Elytrigia* plants had expanded and gained on average 50% more shoots. In contrast, we found that not all *Ammophila* plants had recovered yet or had only just started expanding beyond their initial size [152.6 ± 10.1% (*Elytrigia*), 102.8 ± 8.9% (*Ammophila*); *F*_1,72_ = 18.16; *P *< 0.001; Fig. 2b]. Again, we found a clear position effect with the *Ammophila* plants growing on the higher end of the gradient (foredune) exhibiting less recovery (69.5 ± 4.3%) compared to the expanding plants on the embryonic dunes (136.0 ± 13.9%). For *Elytrigia* there were little differences between the plants on the higher end of their spatial distribution in the embryonic dunes (141.9 ± 10.8%) compared to the plants living on the beach (163.4 ± 16.9%) (*F*_1,72_ = 6.71; *P *= 0.011). Strikingly, no significant main or interactive effects of clonal integration treatment were found (*F*_1,72_ = 0.09; *P *= 0.753).

### Nutrient levels in soil and plant

**Fig. 3:**
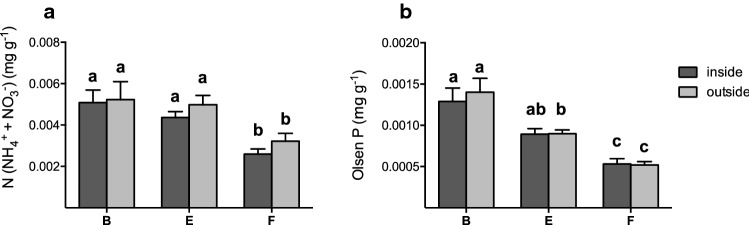
Soil nutrient levels of **a** plant-available nitrogen and **b** plant-available phosphorus, both inside (light grey) and at 50 cm distance of the plants (dark grey) along the successive gradient from beach (B) to embryonic dune (E) and foredune (F). Errors bars represent + SE. Letters depict post hoc (Tukey) grouping (*P *< 0.05)

Soil nutrient (N, P) levels were generally very low and we found no differences between plant-available N or P levels from sediment samples taken within the vegetation patch compared to samples taken at 50 cm distance from the vegetation (N: *F*_1,106_ = 0.32; *P *= 0.572; P: *F*_1,106_ = 0.73; *P *= 0.719; Fig. 3). Plant-available N levels decreased over the biogeomorphic succession gradient and were nearly twice as high on the beach and the embryonic dunes compared to the higher foredunes [0.0052 mg g^−1^ (B&E) vs. 0.0029 mg g^−1^ (F); *F*_2,36_ = 11.93; *P *< 0.001; Fig. 3a]. Similar to N, plant-available P decreased with distance from the sea and was highest at the beach (0.0013 mg g^−1^) and half as much at the foredunes (0.0005 mg g^−1^) (*F*_1,38_ = 32.0; *P *< 0.001; Fig. 3b).

**Fig. 4:**
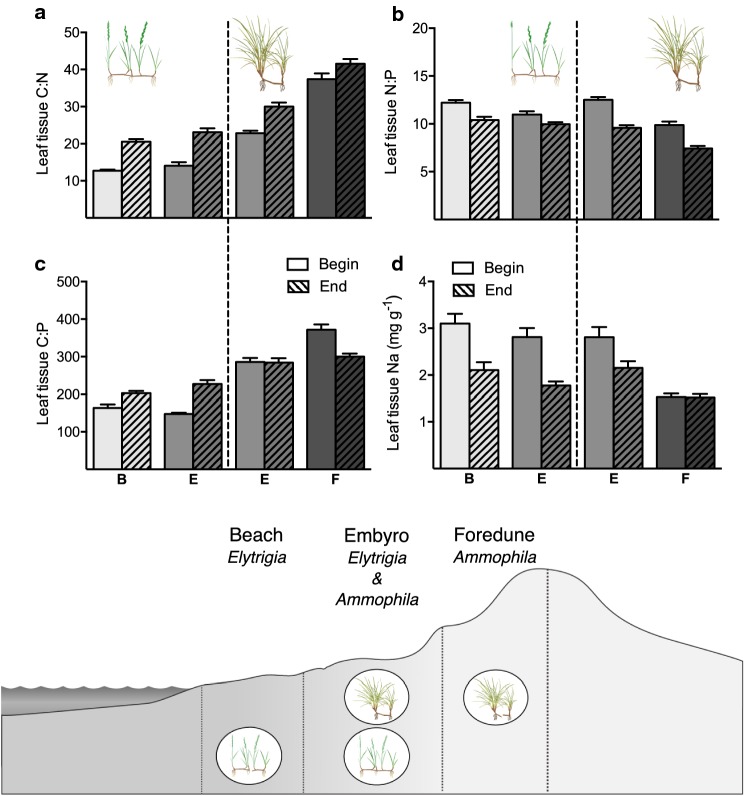
Foliar nutrient ratios for both beach grasses (*Elytrigia*) and (*Ammophila*) at the start and the end of the experiment along the successive biogeomorphic gradient from beach (B) to embryonic dune (E) and foredune (F). **a** C:N ratios in g g^−1^, **b** N:P ratios in g g^−1^, **c** C:P ratios in g g^−1^ and **d** Na contents in mg g^−1^. Error bars represent + SE

C:N leaf tissue ratios were lower for *Elytrigia* compared to *Ammophila* [17.9 ± 0.5 g g^−1^ (*Elytrigia)* vs. 33.2 ± 0.8 g g^−1^ (*Ammophila*); *F*_1,74_ = 332.7; *P *< 0.001; Fig. 4a]. Furthermore, we found a strong interaction between species and their position along the biogeomorphic gradient, as we observed no significant differences between the two *Elytrigia* stages and a sharp increase in C:N ratio for *Ammophila* further up the gradient (+ 1.4 from EB to EE and + 14.6 from AE to AF; *F*_1,74_ = 18.9; *P *< 0.001). At the end of the experiment, the C:N ratio was higher for all treatment levels with no significant interactions (*F*_1,220_ = 177.9; *P *< 0.001). By comparing the nitrogen content of shoots on either end of the clonal network, we found very little variation between plant parts at the start of the experiment, but nitrogen content varied more at the end of the growing season [0.03 (start) vs. 0.10 (end); *F*_1,146_ = 17.48; *P* < 0.001; Online Resource 1 Figure S1a,b, Table S1]. We found no significant effects or interactions of neither species identity nor clonal integration treatment level (Online Resource 1 Table S1).

Similar to foliar C:N ratios, the C:P ratios of *Elytrigia* shoots were on average lower than the *Ammophila* foliar ratios [187.8 ± 4.7 g g^−1^ (*Elytrigia*) and 309.7 ± 6.3 g g^−1^ (*Ammophila*); *F*_1,76_ = 253.9; *P *< 0.001; Fig. 4c]. Moreover, we found an interaction effect of both position on the biogeomorphic gradient (lower vs. higher end), species identity and the time of measurement, which indicates that the change in C:P ratio for both species over time differed based on their position on the biogeomorphic gradient. This resulted in an increase in C:P ratio for *Elytrigia* on both the lower (+ 40 from start to end at beach) and higher (+ 80 from start to end at embryonic dunes) ends of its spatial distribution, whereas the C:P ratio of *Ammophila* did not change at the lower end of its distribution (− 1.5 from start to end at embryonic dunes) but showed a decrease at the highest point (− 71 from start to end at foredunes) (*F*_1,215_ = 12.68; *P *< 0.001). The foliar N:P ratios reflected the C:N and C:P ratios. Again, the species differed with *Ammophila* having an on average lower N:P ratio than *Elytrigia* [9.7 ± 0.2 g g^−1^ (*Ammophila*) vs. 10.9 ± 0.2 g g^−1^ (*Elytrigia*); *F*_*−*1,76_ = 14.5; *P* < 0.001; Fig. 4b]. We found an interaction effect between species and time of measurement (*F*_−1, 214_ = 11.4, *P *< 0.001) and between species and their relative position on the biogeomorphic gradient (*F*_1,76_ = 8.0; *P *= 0.006) with steeper decline in N:P ratios reported for *Ammophila* between the start and the end of the experiment than *Elytrigia* [− 2.9 (AE) and − 2.5 (AF) vs. − 1.8 (EB) and − 1.0 (EE)]. The N:P ratios at the end of the experiment were highest for *Elytrigia* on the beach and lowest for *Ammophila* on the foredune [10.4 ± 0.4 (EB) vs. 7.4 ± 0.3 (AF)].

Lastly, the Na content of the leaves differed between species with higher Na values for *Elytrigia* than *Ammophila* [2.43 ± 0.09 mg g^−1^ (*Elytrigia*) vs. 1.99 ± 0.08 mg g^−1^ (*Ammophila*); *F*_1,76_ = 11.92; *P *< 0.001; Fig. 4d]. The Na content decreased over the biogeomorphic gradient with the highest overall values in the leaves of *Elytrigia* on the beach front (2.60 ± 0.14 mg g^−1^) and the lowest values for *Ammophila* at the dune crest (1.53 ± 0.05 mg g^−1^) (*F*_1,76_ = 22.87; *P *< 0.001). Furthermore, we found an interaction effect between the species and the time of measurement as the foliar Na values for *Elytrigia* strongly decreased over time (− 1.03 from start experiment to end) and the Na leaf content of *Ammophila* responded less strongly (− 0.34 from start to end) (*F*_1,229_ = 45.97; *P *< 0.001).

## Discussion

We hypothesized that landscape-forming plants would rely on clonal integration in the pioneer stage of biogeomorphic succession where physical stress is high, and that this dependency would be less important in later stages where conditions are more benign through biophysical modifications of the landscape. Although previous studies from similar ecosystem types (e.g. salt marshes and inland dunes) reported beneficial effects of clonal integration in overcoming physical stress (Pennings and Callaway 2000; Yu et al. 2004; Xiao et al. 2010), surprisingly, our experiment conducted on a Western European coastal dune system did not reveal any differences in recovery and expansion rates between connected and severed clonal individuals. Instead, we found the response to physical stress to differ greatly between the two species investigated in our experiment and their relative position on the biogeomorphic gradient. The pioneer species, *Elytrigia*, showed a high resilience regardless of its habitat (beach or embryonic dune). In contrast, the later successional species, *Ammophila*, exhibited a high resilience on the lower end of its spatial distribution (embryonic dunes), but did not fully recover on the foredunes (Fig. 2), possibly as a result of nutrient deprivation. Our results indicate that although the development of high dune landscapes may increase the resistance of beach grasses to the physical and physiological challenges of coastal flooding events (Baye 1990; Durán and Moore 2013), the reduced nutrient input may negatively impact their resilience to severe disturbance. In addition, our experimental results suggest that clonal integration plays a negligible role in determining the resilience of dune grass species independent of their successive stage. Although important, we stress that these findings are currently based on experiments conducted at a single location over the course of one growing season. Hence, to understand how common our findings are for these dune grass species or landscape-forming plants in general, we argue that similar field studies across a diverse set of systems with different species and conditions are required.

### Nutrient availability in coastal dunes

Harsh environmental conditions in sandy beach environments (e.g. wave impact, salinity, burial, low freshwater and nutrient availability) hamper plant establishment and outgrowth (Maun 1994). Through biophysical feedbacks, beach grasses can escape the detrimental effects of seawater flooding and enhance freshwater retention, but in turn they have to cope with an increase in other stressors such as sand burial and nutrient limitation (Maun 1998; Dugan et al. 2011; Feagin et al. 2015; Brown and Zinnert 2018). In our dissipative coastal dune system, we found nutrient levels to be generally very low and to exhibit little spatial variability and no relation to vegetation presence (Fig. 2). Furthermore, the lower influence of seawater intrusion over the dune biogeomorphic succession gradient (as reflected by the decrease in Na content; Fig. 4d), with increasing elevation and distance to the sea (Fig. 1), led to a decreased marine nutrient input, increasing foliar C:N and C:P ratios (Hannan et al. 2007; Dugan et al. 2011). Although the foliar P levels also decreased over the gradient (Fig. 4c), previous studies have found that especially N availability strongly affects the growth of beach grasses (Willis 1965; Kooijman et al. 1998; Kooijman and Besse 2002; Heyel and Day 2006). The occurrence of N limitation for both grasses is supported by relatively low N:P ratios (Güsewell 2004) that were lowest for both plants species at the higher end of their spatial distribution and decreased over time [at end experiment: 9.9 g g^−1^ (*Elytrigia* embryonic dune) vs. 7.4 g g^−1^ (*Ammophila* foredune); Fig. 4a]. The lower C:N and C:P ratios of *Elytrigia* compared to *Ammophila* (~ 65%) at the embryonic dunes, where both species were co-occurring, indicates a higher nutrient use efficiency for the pioneer species (Fig. 4a, c). Although previous studies have found that the contribution of biological N fixation (through bacteria or fungi) becomes more important over the succession gradient and can greatly enhance N availability (Dalton et al. 2004; Eduardo et al. 2006; Jones et al. 2008), we only observed a decrease of both the absolute and the relative (with respect to P) N availability with increasing distance from sea. These findings suggest that seawater flooding is an important nutrient source in these coastal dune systems. Nutrient limitation at higher elevations may not only decrease the resilience of dune grass species to severe disturbances (e.g. storm damage or grazing), but may also enhance their susceptibility to pathogens, such as root-feeding nematodes, or interact with other stressors of coastal dune systems such as drought (Huber and Watson 1974; Park 1990; van der Putten et al. 1993; Dorras 2008; Mullins et al. 2019).

### Mechanisms to cope with low nutrient availability

To cope with the nutrient-poor conditions prevailing in the higher dune areas, *Ammophila* can recycle its own plant material through a litter-decomposition feedback. A foliar C:N ratio of > 30 makes the plants poorly degradable and leaves generally stay on the plant for 2 years before falling off (Kooijman and Besse 2002). By accumulating a high quantity of slowly decomposing plant material, with a very high N-mineralization per unit litter, *Ammophila* can normally regenerate sufficient nutrients for maintenance and further growth (Kooijman and Besse 2002). As extreme high storm surges may remove all standing biomass, this may inhibit this litter-decomposition feedback and lower the resilience of the *Ammophila* plants on the foredune. The especially very low N:P ratio of the plants on the foredune at the end of the biomass-removal experiment (~ 7.4) indicates severe N deficiency (Kooijman et al. 1998; Güsewell 2004). In contrast, the *Elytrigia* and *Ammophila* plants growing in the lower regions were able to maintain their N:P ratio around ~ 10 and showed no clear response to reduced N input. Overall, we found that in the early successional phases of coastal dune development (from beach to primary foredune), marine nutrient input strongly contributes to nutrient availability and increasing N limitation eventually reduces the resilience of *Ammophila* plants on the primary dune crest.

### The importance of clonal integration in beach systems

Although most studies in similar ecosystem types (e.g. inland dunes, salt marshes) have found that clonal integration helps clonal species overcome the hostility of their environment (Evans 1991; Dong and Alaten 1999; Yu et al. 2004; Liu et al. 2009; Pennings and Callaway 2000), some studies reported no or context-dependent effects of clonal integration (Wang et al. 2004; Hellström et al. 2006). In our experiment in a single location on a Western European beach system, we found no effects of rhizome severing on the recovery and expansion rate of our plants (Fig. 1) or on the nitrogen content (Online Resource 1, Figure S1a, b).

Most studies that reported a positive or facilitative effect of clonal integration subjected the plants to heterogeneous nutrient or stress levels (Liu et al. 2016). In contrast, in our study system we found very little spatial heterogeneity in soil nutrient levels, which is probably related to the very low influx of nutrient-rich wrack on this wide dissipative beach. The low spatial variability in nutrient levels limits the significance of foraging for nutrients and may potentially explain the absent role of clonal integration in controlling beach grass resilience. As wrack subsidies and thus nutrient influxes are often spatially heterogeneously distributed depending on the topology of the beach, the nature of the wrack material and tidal characteristics (Orr et al. 2005), we expect a larger contribution of clonal integration on beaches with significant wrack deposition. To further elucidate the ecological significance of clonal integration for dune-building grasses, we suggest performing similar experiments under various marine subsidy conditions (low vs. high wrack) and with a high or low spatial variability in nutrient conditions.

### Escaping physical stress to accept hunger

We found the resilience of beach grasses to severe plant-level disturbance to be largely dependent on their position on the biogeomorphic gradient. For the pioneer species, *Elytrigia*, we observed no differences in resilience, although the recovery rate after 21 days was slightly higher at the higher end of its distribution at the embryonic dunes (Fig. 2a). Since the plants growing on the beach were very small at the beginning of the growing season [av. 15 shoots (beach) vs. 43 shoots (embryonic dune)], we expect their initial lag in recovery to be the result of a lower initial biomass and reserves (Harris and Davy 1986b). Although the *Elytrigia* plants on the beach quickly recovered and showed 60% more shoots at the end of the experiment, we expect that storm surges in the winter season will heavily impact these plants contributing to their relatively small size at the start of the season. The high recovery and expansion rate of *Elytrigia*, probably also related to its high nutrient use efficiency, likely form the basis for its capacity to colonize these highly dynamic areas. By accumulating and stabilizing wind-blown sand, the species can eventually create a more stable environment. Additionally, the dispersed clonal expansion strategy of *Elytrigia* leads to the formation of low, but wide dune profiles which get flooded a few times a year, providing the delivery of marine nutrient input. For the later successional species, *Ammophila*, we observed no position-dependent differences in plant size at the beginning of the experiment [av. 90 shoots (embryonic dune) vs. 83 shoots (foredune)], but we did find clear differences in its resilience to severe physical stress. The patchy and dense clonal expansion strategy of *Ammophila* (Reijers et al. 2019a), in combination with the ability to grow vertically expanding rhizomes, leads to a higher and steeper dune profile, which eventually allows the species to escape high storm surges. The litter-nutrient feedback would normally allow *Ammophila* to grow and expand in these nutrient-poor environments. However, when a large storm hits the foredune and removes all standing biomass (as we simulated with our clipping treatment), we predict nutrient deprivation to lead to a very low recovery potential.

### Clonality and vegetated coastal ecosystem

Many vegetated coastal ecosystems, including seagrass meadows, salt marshes and coastal dunes, are formed by clonally expanding plants (Kendrick et al. 2005; Hacker et al. 2012; Bouma et al. 2013). Clonal expansion allows plants to effectively colonize a certain area and to manipulate their spatial shoot organization, thereby actively facilitating further landscape formation through biogeomorphic feedbacks (Schwarz et al. 2018; Reijers et al. 2019a). We here show that, at least in homogeneously nutrient-poor environments as our beach system, the development of a clonal network does not necessarily increase nutrient availability, by foraging for nutrients or translocating nutrients through the rhizomal network. Although nitrogen content was very similar between plant parts at the start of the growing season, at the end of experimental period the variation increased for both species independent of integration treatment. This may indicate that for these dune grasses, the ecological significance of clonal integration is limited during the growing season. However, as our experiment was conducted on a single location, we do not yet know whether our results reflect the general behaviour of these dune grasses. In fact, other studies have reported positive effects of clonal integration in other dune (Yu et al. 2004) or coastal ecosystems such as salt marshes (Pennings and Callaway 2000) and seagrass meadows (Marbà et al. 2002). However, as these studies were also conducted in single habitats, it is currently not possible to draw a general conclusion on how and when landscape-forming plants in natural environments benefit from clonal integration. It does, however, indicate that both the potential to translocate nutrients and the effect of this trait are likely context dependent and may differ greatly depending on the clonal network architecture of the species, resource availability and spatio-temporal environmental heterogeneity (Liu et al. 2016). We therefore emphasize the need to integrate research on clonality and habitat modification (Brooker 2017) in natural settings to better understand the main processes determining the resilience and dynamics of these important feedback-dependent ecosystems.

## Electronic supplementary material

Below is the link to the electronic supplementary material.

Supplementary material 1 (PDF 138 kb)

## Data Availability

Data are available from the Data Archiving and Networked Services (DANS) EASY: 10.17026/dans-zq6-pzdk (Reijers et al. 2019b).
