# Supplementary material for: Resilience of beach grasses along a biogeomorphic successive gradient: resource availability vs. clonal integration
Source: Oecologia. 2019 Dec 4;192(1):201–12. doi: 10.1007/s00442-019-04568-w (PMC6974500; doi:10.1007/s00442-019-04568-w)
Supplement: Supplementary file 1 — Supplementary material 1 (PDF 138 kb) [file 442_2019_4568_MOESM1_ESM.pdf]

Supplementary information

Resilience of beach grasses along a biogeomorphic successive gradient: resource availability versus clonal integration

Valérie C. Reijers, Carlijn Lammers, Anne J.A. de Rond, Sean C.S. Hoetjes, Leon P.M. Lamers & Tjisse van der Heide

Contact information corresponding author:

Valérie C. Reijers

Heyendaalseweg 135, 6525 AJ Nijmegen, the Netherlands

v.reijers@science.ru.nl

+31 644890397

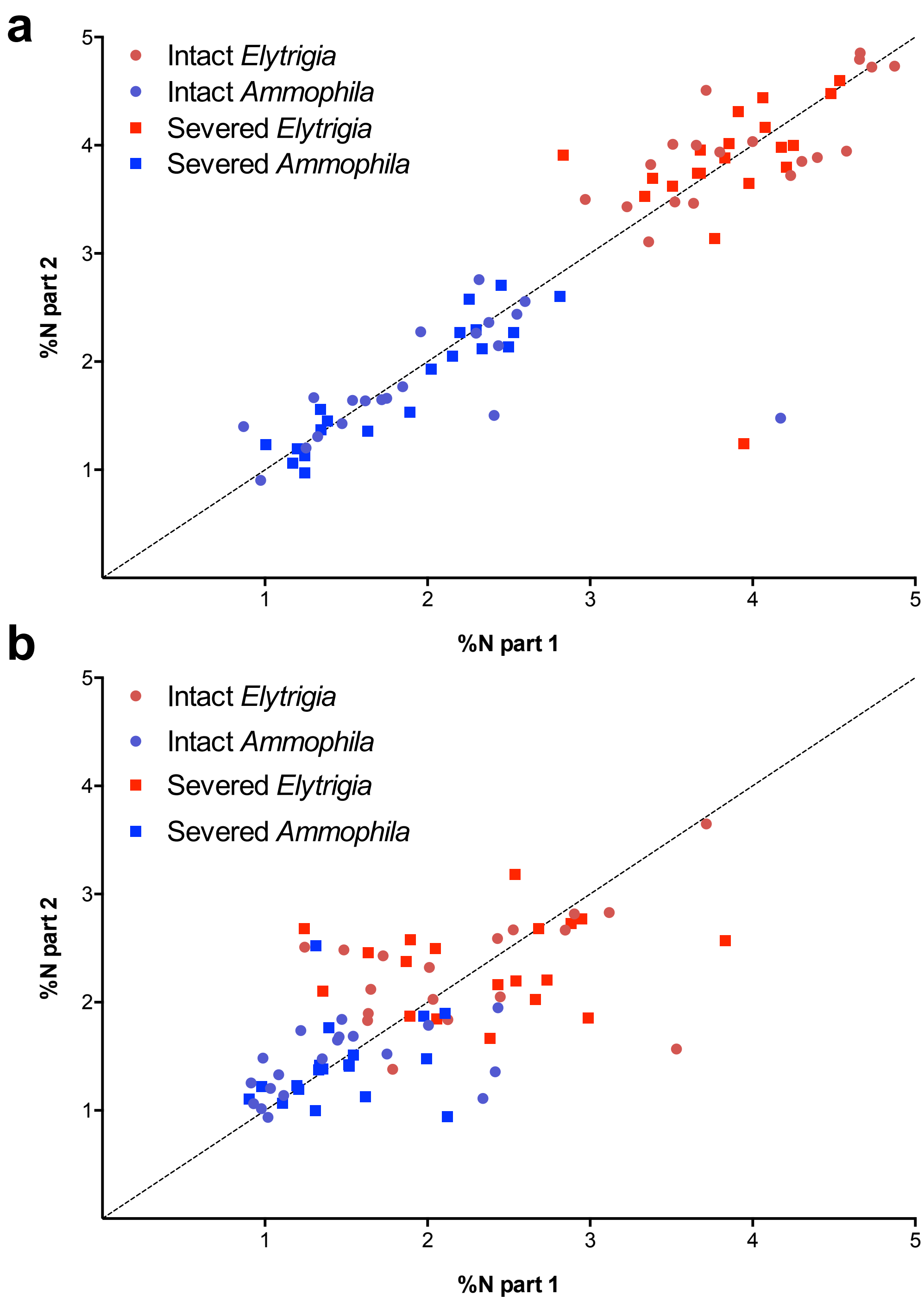

Figure S1: Relation in foliar nitrogen content (%) between both parts of the clonal individual for both *Elytrigia* (red markers) and *Ammophila* (blue markers) at the start (**a**) and at the end of the experiment (**b**). The dotted line indicates the 1:1 relation between both plant parts.

Table S1: Statistical outcome of the three-way ANOVA on the differences in N content between the two plant parts based on species identity, integration treatment and timepoint.

Effect	Df	F	P
Species	1	0.034	0.855
Treatment	1	0.155	0.695
Time	1	17.479	<0.001 ***
Spec*Treat	1	0.747	0.389
Spec*Time	1	0.947	0.332
Treat*Time	1	0.034	0.854
Spec*Treat*Time	1	1.086	0.299
Residuals	146		
